# Hydrothermal carbonization of vegetable-tanned leather shavings (HTC-VTS) for environmental remediation: optimization of process conditions

**DOI:** 10.1098/rsos.230302

**Published:** 2023-10-18

**Authors:** Baissassou Debina, Abdelaziz Baçaoui, Aymard Didier Tamafo Fouégué, Daouda Kouotou, Abdoul Ntieche Rahman, Abdelrani Yaacoubi, Loura Benguellah Benoît

**Affiliations:** ^1^ Department of Chemistry, Faculty of Science, The University of Maroua, PO Box 814, Maroua, Cameroon; ^2^ Department of Chemistry, Faculty of Science Semlalia, University of Cady Ayyad, Marrakech, Morocco; ^3^ Department of Chemistry, Higher Teacher Training College Bertoua, The University of Bertoua, PO Box 652, Bertoua, Cameroon; ^4^ Physical and Theoretical Chemistry Laboratory, Department of Inorganic Chemistry, Faculty of Science, University of Yaoundé I, Yaoundé, Cameroon

**Keywords:** biomass, environmental remediation, hydrothermal carbonization, hydrochar, response surface methodology, vegetable-tanned leather shavings

## Abstract

Herein, the response surface methodology (RSM) has been used to study simultaneously the effects of carbonization temperature, residence time and moisture content on the activated hydrochar preparation-based vegetable-tanned leather shavings (VTS) using hydrothermal carbonization method (HTC). Owing to the desirability chosen, three responses were analysed, namely: the hydrochar yield, iodine and methylene blue numbers. The analysis of experimental results revealed that the hydrochar yield was decreased with increase in carbonization temperature which led to micropores formation inside the hydrochar network. The optimal preparation conditions retained were: 83.10%, 390.44 mg g^−1^ and 259.63 mg g^−1^ for the hydrochar yield, iodine and methylene blue number respectively. The hydrochar micrograph showed the presence of external pores, whereas the FTIR analysis recorded the presence of acidic functional groups found on hydrochar surface. The findings revealed that the VTS is a good precursor for the hydrochar preparation useful in the removal of organic and inorganic pollutants in aqueous media.

## 1. Introduction

Nowadays, the research of outstanding porous materials for the removal of various pollutants from liquid or gaseous effluents using adsorption technique is a matter of global concern. Some primary materials such as biomass wastes [1–4], organic polymers [5–7], clays [8], silica gel [9], activated alumina [10], zeolites [11–13], industrial and municipal wastes [14] have been studied and used as precursors for the porous materials preparation. Their high adsorption capacity, high efficiency for adsorbing substances at low concentrations, high selectivity, easy regeneration and low cost are the required criteria for the good adsorbents production. In this light, the carbon materials-based biomass wastes have shown promising application for the sorption utilities [1–3]. However, the problem remains that there is neither general satisfaction nor a suitable process for the production of valuable carbonaceous materials from the raw lignocellulosic biomass wastes to date.

It is in this perspective that, the hydrothermal carbonization (HTC) process could be the cornerstone and a promising method to be exploited. The HTC has several advantages; just to cite one: many organic materials are found in aqueous medium and which obviously have a high water content, make the HTC process ideally suitable for the carbonization of such material. Since no drying process is required, the HTC method offers a 50% energy saving compared with other methods which require drying beforehand [15–20]. The HTC equally enables the washing of inorganic elemental compositions into the liquid phase and reduces significantly the ash content. In addition, it has the advantage of maintaining significant chemical surface functionality in the hydrochar, by developing oxygen and nitrogen functional groups, owing to its lower operating temperature [4–7]. However, hydrothermal carbonization presents as main disadvantage the high set-up requirements (energy and installation costs) for the equipment [21]. Solid discharge from tanneries are found everywhere in the environment, principally in an aqueous medium with a highly humid form. The tannery industries discharge into the environment huge quantity of solid waste and wastewaters. In 2009, it was reported that the average wastewater and solid waste discharges into the environment were 1.5 × 10^10^ m^3^ and 6 × 10^9^ kg, respectively [21]. It has been shown in our previous work [22] that transforming solid waste derived from the tannery industries into adsorbent for the removal of contaminants is an interesting alternative for environmental remediation and for the production of low-cost materials.

The composition of the liquid part from tanneries discharges is linked to the process used in tanning skins, as it consists essentially of a mixture of chemicals such as sodium hydroxide, sodium hypochlorite, potassium dichromate, lime, chlorides, sulfuric acid, formic acid, surfactants, sodium sulfide, sodium and ammonium salts, chromium (III) and dyes [20]. The composition of the solid part consists mainly of hair, fragments of flesh and skins belonging to slaughtered animals like sheep, goats or cows [22]. The city of Marrakech in Morocco is a region where a huge amount of leather material is produced because of the existence of many local and traditional tannery industries. Here, the problem is not the presence of these industries of leather materials, but the main problem is the destination taken by the discharge, as well as its management. The environmental effects of the tanning process are significant and need to be addressed. Despite the socio-economic impact of the tanning industries through job creation and income generation, the population around the tanning industries are exposed to pollution resulting from this activity.

In order to address the issue of pollution and allow the social integration of the workers, it is important to collect these solid wastes (vegetable-tanned leather shavings) from tanning industries and give them an added value by transforming them directly into carbonaceous materials by HTC method [20–23]. To achieve this goal, optimization through the response surface methodology involving screening of parameters has been adopted. The main factors such as the residence time, humidity rate and final temperature of the carbonization are important parameters in optimizing the preparation method of hydrochar. Owing to the fact that the carbonaceous materials in quantity and quality are needed and additionally, the hydrochar material prepared will be used to remove harmful substances from drinking water, domains of variation of predictive variables such as hydrochar yield, Y_ld_ (%); iodine number, ION (mg g^−1^); and methylene blue number, MBN (mg g^−1^) will be studied in order to obtain the hydrochar characteristics required.

## 2. Material and methods

### 2.1. Preparation of raw material

The vegetable-tanned leather shavings (VTS) used in this study were obtained from a traditional tannery in Marrakech, Morocco. Prior to the experiments, the VTS wastes were cut into small pieces, washed with distilled water and then put under stirring overnight. Thereafter, it was mixed with a solution of acetic acid (16.0 g l^−1^) thrice to remove mineral substances. The pH value of the solid waste was adjusted between 4.8 and 5.0 with an acetic-sodium acetate buffer solution. The mixture obtained was dehydrated using absolute ethyl alcohol and dried in vacuum to a moisture content less than 10.0%.

The VTS sample before the HTC was characterized by thermogravimetric analysis using a Perkin-Elmer DTA-TGA analyser, which was carried out in inert atmosphere (100 ml min^−1^) at different heating rate (5; 10; 15; 20 and 50°C min^−1^) and at temperature between 18°C and 800°C.

### 2.2. Hydrothermal carbonization process of biomass

An exact amount of 15.5 mg of VTS with different moisture contents were loaded into a reactor associated with an autoclave that was heated from room temperature (18°C) up to the target temperature set under an N_2_ atmosphere at the heating rate of 5°C min^−1^. At each final temperature and residence time, the oven was turned off and allowed to cool to room temperature inside the autoclave. The resulting hydrochar was labelled as VTS-HTC and was weighed following equation (2.1). The VTS-HTC was then oven dried at 105°C for 24 h.

2.1 $$\mathrm{VTS}-\mathrm{HTC}\;\mathrm{mass}\;\mathrm{yield}(\text{\%})=\frac{m_{\mathrm{hydochar}}}{{\mathrm{mass}}_{\mathrm{VTS}}}\times 100.$$

$m_{\mathrm{hydochar}}$ in equation (2.1) is the mass of the hydrochar after being dried and ${\mathrm{mass}}_{\mathrm{VTS}}$ is the mass of the raw material with moisture. After the hydrocarbonization process, about 5.0 to 6.0 g of the hydrochar was subjected to physical activation with steam (0.13 ml min^−1^) in a furnace by heating the reactor from room temperature up to 850°C at the heating rate of 10°C min^−1^ for 2 h of residence time.

The demineralization of activated VTS-HTC (hereafter referred as activated hydrochar or simply AH) obtained was done to decrease its inorganic content by HCl treatment and washed with hot water, followed by washing with cold water until no chloride ions could be detected (by testing with AgNO_3_ solution) and finally dried.

#### 2.2.1. Optimization process

**Figure 1.**
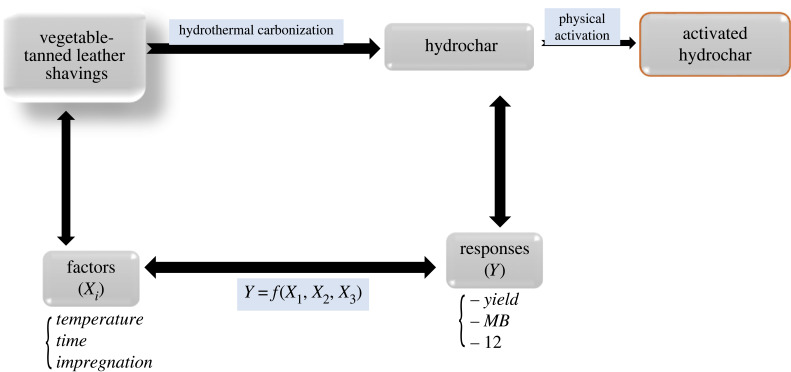
Preparation process of hydrochar by the design of experiment method from vegetable-tanned leather shavings.

The hydrothermal carbonization process parameters were studied using the response surface methodology (RSM) (figure 1). The RSM is a statistical technique that is useful for modelling and analysing problem where a response of interest is influenced by several variables [24]. RSM aims at reducing the number of experiments to be performed, while simultaneously studying the effects of several factors, as well as helping to analyse interactions between the parameters studied [24–26].

The most influential experimental factors on the final characteristics of carbonaceous material obtained from HTC are the carbonization temperature (*X_1_*), the residence time (*X_2_*) and the moisture content (*X_3_*). The three responses analysed were hydrochar yield (*Y*_1_), iodine number, (*Y*_2_) and methylene blue number (*Y*_3_). The Doehlert's experimental matrix and the corresponding experimental conditions of the responses *Y*_1_, *Y*_2_ and *Y*_3_ are given in table 1. Each response was used to develop a model correlating the responses to the three coded factors using a polynomial equation as follows:

2.2 $$Y_{i}=b_{0}+b_{1}X_{1}+b_{2}X_{2}+b_{3}X_{3}+b_{11}X_{1}^{2}+b_{22}X_{2}^{2}+b_{33}X_{3}^{2}+b_{12}X_{1}X_{2}+b_{13}X_{1}X_{3}+b_{23}X_{2}X_{3\;}+\mathrm{residual}.$$

In equation (2.2), *Y_i_* is the predicted response, *b_0_* is a constant coefficient, *b_i_* is a linear coefficient; *b_ii_* is a quadratic coefficient, *b_ij_* is an interaction coefficient; *X*_1_, *X*_2_ and *X*_3_, are the coded values of the respective factors.

**Table 1.** Doehlert's experimental matrix, the corresponding experimental conditions and responses. *Y*_1_: total yield (%); *Y*_2_: iodine adsorption capacity (mg g^−1^); *Y*_3_: methylene blue adsorption capacity (mg g^−1^)

Exp no	rand	temperature (*X*_1_)	residence time (*X*_2_)	humidity content (*X*_3_)	*Y*_1_	*Y*_2_	*Y*_3_
1	1	290	75	700	5500	27 919	240 00
2	2	190	75	700	8500	30 139	260 00
3	3	265	110	700	5700	26 000	5000
4	4	215	40	700	7500	21 000	9600
5	5	265	40	700	6000	31 700	18 000
6	6	215	110	700	7200	41 200	12 000
7	7	265	87	782	5517	22 203	11 000
8	8	215	63	618	7800	30 726	20 000
9	9	265	63	618	6140	28 550	23 000
10	10	240	98	618	6800	34 899	18 000
11	11	215	87	782	7386	25 381	14 000
12	12	240	52	782	6500	25 700	19 800
13	13	240	75	700	6600	25 226	6000
14	14	240	75	700	6473	26 967	8000
15	15	240	75	700	6560	22 678	8000
16	16	240	75	700	6500	27 919	7000
17	17	240	75	700	6700	31 726	9 500

The number of experiments required (*N*) is given by

2.3 $$N=k^{2}+k+C.$$

Where, *k* is the number of variables and *C* is the number of centre points. In the present work, *k* is equal to 3 with five experiments at the centre of the studied domain. These experiments at the centre of the experimental matrix are used to determine the experimental error and check the reproducibility of the results obtained. Therefore, the matrix has nine experiments.

### 2.3. Carbonaceous material characterization

The activated hydrochar prepared at the optimal condition was characterized by various physico-chemical methods. The AH adsorptive property in liquid phase was determined by iodine and methylene blue adsorption capacities. To determine the MBN, a solution of methylene blue (MB) was prepared by dissolving 600 mg in 2.0 l of distilled water. The mixture was stirred for 12.0 h and then filtered to remove the undissolved particles. An amount of 10.0 mg aliquot of the prepared AH was weighed into a bottle and 100.0 ml of the MB solution was added to the bottle and shaken at the rate of 200 r.p.m. at room temperature for 4.0 h. A control was treated in a similar manner but for the fact that it did not contain AH. After agitation, the mixture was filtered; an aliquot of 1.0 ml was measured and diluted with 100 ml of distilled water. The absorbance was then measured using a UV-visible spectrophotometer (Jenway 7310, Jenway, Staffordshire, UK). The concentration at equilibrium is deduced using Beer's Law, then the adsorbed quantity is determined by the following equation:

2.4 $$Q_{\mathrm{ads}}=\frac{\left(C_{0}-C_{r}\right)}{m}\times V.$$

*Q*_ads_ is the quantity of MB adsorbed per unit mass of AH (in mg g^−1^), *C*_0_ is the initial concentration of MB (mg l^−1^), *C_r_* is the residual concentration of MB (mg l^−1^), *V* is the volume of the solution (*L*) and *m* is the mass of the adsorbent (g).

The ION of AH was obtained on the basis of the American Standard Test Method (ASTM) by titration with pentahydrated sodium thiosulfate. The concentration of iodine solution adsorbed was thus calculated from the total volume of sodium thiosulfate used and volume dilution factor (equation (2.4)).

The textural characteristics of the activated hydrochar were obtained using a chemisorption and physisorption surface area analyser (Micromeritics TriStar 3000). The AH was out-gassed in vacuum at 353 K and 5 µm Hg during 6 h prior to measurement. The specific surface area of AH was estimated by Brunauer–Emmett–Teller (BET) model, and the micropores volume and external surface area were obtained by *t*-plot method using Harkins–Jura equation (equation (2.5)) for calculation of adsorbed layer thickness.

2.5 $$t(\mathrm{nm})={\left[\frac{0.1399}{0.034-\log(P/Po)}\right]}^{0.5}.$$

The surface functional groups of the obtained samples were determined by Fourier transform infrared (FTIR) spectrum using FT-IRSPECTRUM ONE brand, the wave number was found between 450 and 4000 cm^−1^. The surface morphology was investigated using scanning electron microscopy (SEM) (VEGA3 TESCAN). Elemental energy dispersive X-ray (EDX) analysis was done using EDAX TEAM, 125.9 eV of resolution and was applied to investigate the presence and percentage of atoms that made up the AH.

## 3. Results and discussion

### 3.1. Differential thermogravimetric analysis and thermogravimetric analysis of the raw material

**Figure 2.**
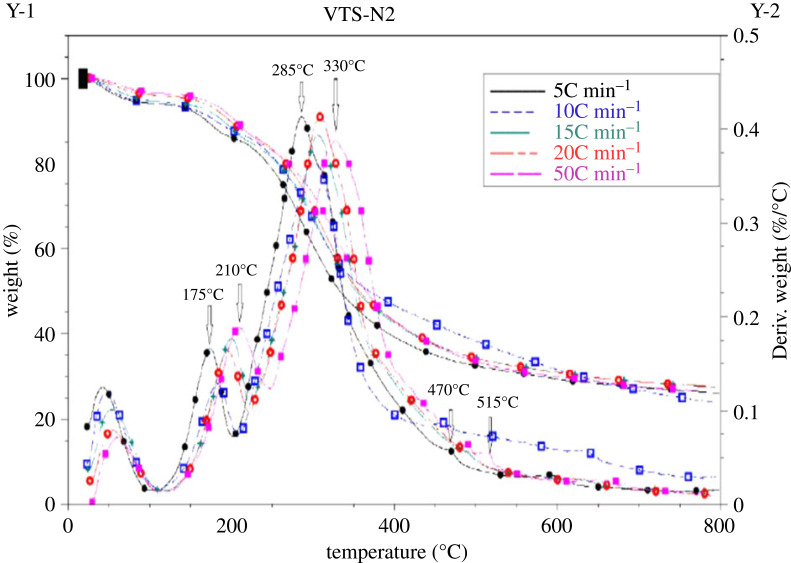
TGA/DTG curves of the vegetable-tanned leather shavings (VTS) at different heating temperatures.

The thermogravimetric analysis (TGA) and differential thermogravimetric analysis (DTG) are used to assess the heat stabilities associated with the mass change between 25 and 900°C (figure 2). At first sight the curves plotted at different heating temperature for the VTS are almost identical and the important changes take place at the same point. It can be seen that there is a first variation between 40.5°C and 210°C corresponding to the decrease in mass of 7.6%, due to a first water departure according to an endothermic reaction (Δ*H* = + 693.5 J g^−1^). The most important stage in weight loss starts from 175.5°C to 631°C; surely due to the removal of volatile organic substances and moisture, this is explained by the appearance of intense peaks at 285°C and 330°C on the DTG-curve, with the weight loss of 52.1%. Beyond the temperature of 600°C, the mass varied slightly depending on the temperature.

### 3.2. Response surface analysis

The responses selected in this work are useful tools to provide important information on the adsorption properties of the activated hydrochar. The hydrochar yield (*Y*_1_) studied here as the percentage of weight loss also known as percentage of burn-off is to predict the porous structure of the prepared hydrochar. The iodine adsorption test (*Y*_2_) indicates the microporosity of carbon material. It indicates the material adsorption affinity for small molecules and finally the methylene blue (*Y*_3_) has an average size representative model of organic pollutants, which is used to evaluate the performance of carbon before its use in water treatment, bleaching of vegetable oils and other uses [26].

By simply solving the equation through the regression method based on the least squares optimization criterion. The values of the coefficients (table 2) and the ANOVA (table 3) of the regression are directly obtained using the new efficient methodology of research using optimal design (NEMROD) software.

**Table 2.** Estimated values of coefficients for hydrochar yield (*Y*_1_), iodine number (*Y*_2_) and methylene blue number (*Y*_3_).

name	hydrochar yield (*Y*_1_)		iodine number (*Y*_2_)		methylene blue number (*Y*_3_)	
	coefficient	significance	coefficient	significance	coefficient	significance
*b*_0_	65.43	<0.01***	78.20	<0.01***	254.32	<0.01***
*b*_1_	−15.40	<0.01***	−3.75	45.6	−16.61	0.217**
*b*_2_	−1.62	0.381**	−36.81	0.016***	38.40	<0.01***
*b*_3_	−3.06	0.014***	−36.74	0.017***	−42.63	<0.01***
*b*_11_	4.57	0.025***	171.80	<0.01***	35.97	0.056***
*b*_22_	−0.59		−14.87	9.9	48.58	0.012***
*b*_33_	1.62	2.86	103.47	<0.001***	21.04	0.634**
*b*_12_	0.29	74.8	−92.38	0.01***	−149.5	<0.01***
*b*_13_	−2.36	4.51*	−4.08	74.4	52.89	0.073***
*b*_23_	−0.40	69.3	−80.13	0.04***	−49.74	0.100**

^∗∗∗^ most significant effect, ^∗∗^ less significant effect, ^∗^ no significant effect.

**Table 3.** Analysis of variance of the hydrochar yield (*Y*_1_), the iodine number (*Y*_2_) and the methylene blue number (*Y*_3_).

source of variation	sum of square			degree of freedom			square mean			report			significance		
	*Y*_1_	*Y*_2_	*Y*_3_	*Y*_1_	*Y*_2_	*Y*_3_	*Y*_1_	*Y*_2_	*Y*_3_	*Y*_1_	*Y*_2_	*Y*_3_	*Y*_1_	*Y*_2_	*Y*_3_
regression	1.03369 × 10^3^	7.18666 × 10^4^	3.80950 × 10^4^	9	9	9	1.14854 × 10^2^	7.98517 × 10^3^	4.23278 × 10^3^	200.5044	90.2030	87.8679	<0.01***	<0.01***	<0.01***
residual	4.00918E + 0000	6.19671 × 10^2^	3.37204 × 10^2^	7	7	7	5.72830 × 10^1^	8.85245 × 10^1^	4.81720 × 10^1^						
total	1.03770 × 10^3^	7.24862 × 10^4^	3.84322 × 10^4^	16	16	16									

From the table 2, the polynomials equations of the model are given as follows:

3.1 $$Y_{1}=65.420-15.399X_{1}-1.622X_{2}-3.056X_{3}+4.574X_{1}^{2}-0.593X_{2}^{2}+1.623X_{3}^{2}+0.289X_{1}X_{2}-2.362X_{1}X_{3}-0.402X_{2}X_{3},$$

3.2 $$Y_{2}=78.200-3,750X_{1}-36.807X_{2}-36.742X_{3}+171.800X_{1}^{2}-14.868X_{2}^{2}-103.467X_{3}^{2}-92.379X_{1}X_{2}-4.079X_{1}X_{3}-80.133X_{2}X_{3}$$

3.3 $$\begin{array}{rl} \mathrm{and}\;Y_{3} & =254.322-16.611X_{1}+38.401X_{2}-42.633X_{3}+35.968X_{1}^{2}+48.584X_{2}^{2}+29.037X_{3}^{2}\\ & \;-149.538X_{1}X_{2}+52.892X_{1}X_{3}-49.742X_{2}X_{3}. \end{array}$$

According to equation (3.1), the temperature has the largest influence on the hydrochar yield (*Y*_1_), the effect of temperature is negative (*b*_1_ < 0), indicating that the hydrochar yield drastically decreases (about 15.4%) with increase in temperature. This means that within the chosen hydrocarbonization temperature range, i.e. from room temperature to 290°C, both the water and the volatile compounds are released and cause a decrease in the hydrochar yield. The increase in temperature enables the release of volatiles and causes a decrease in the hydrochar yield. This trend has been observed by other authors [27,28]. The degree of fitness of the model was measured through the regression coefficient *R*^2^. From the above equation, *R*^2^ = 0.996 indicates that 99.6% of the total variation in the hydrochar yield was explained by the fitted model. In addition, the *R*^2^-adjusted coefficient $\left(R_{A}^{2}=0.991\right)$ is also high and close to *R*^2^, confirming that the generated models are accurate [29].

The predicted equation describing adsorption of iodine number (*Y*_2_) shows that all coefficients' values except the square variable of temperature are negative, showing their antagonist effects on the iodine adsorption by the prepared hydrochar. Increasing the residence time with high moisture content adversely affects the micropores formation into the hydrochar structure. Furthermore, the quadratic term coefficients related to temperature are positive and higher, indicating a particular impact of the temperature on the micropore volume obtained. The correlation coefficients (*R*^2^) of the response *Y*_2_ is 0.991 and the *R*^2^-adjusted coefficient is 0.980, which indicates a good agreement between the experimental and predicted values.

The temperature, residence time and humidity have significant effects on the predicted equation (*Y*_3_) describing methylene blue adsorption, indicating that the variables used for the hydrothermal carbonization are favourable for the production of hydrocarbon better adapted for the adsorption of large molecule. The linear term coefficients related to the residence time in the equation model of methylene blue (*Y*_3_) is positive showing its significant effect (*p*-value < 0.01***) on the MB adsorption; increasing the residence time enlarges pores inside the hydrochar and promotes the formation of mesopores or external pores, while the carbonization temperature and humidity have an antagonistic effect on the methylene blue adsorption. In addition, the interaction term coefficient $\left(|b_{12}|=149.\;54\right)$ shows that the combined effect between temperature and residence time of carbonization enhances the MB adsorption.

### 3.3 Optimization of VTS-HTC preparation conditions

From the established model, the optimal values can determine the operating parameters leading to the maximum yield of hydrochar preparation by HTC. This is performed either by plotting the iso response curves and response surfaces or by solving the model equation.

#### 3.3.1. The iso response curves and response surfaces

**Figure 3.**
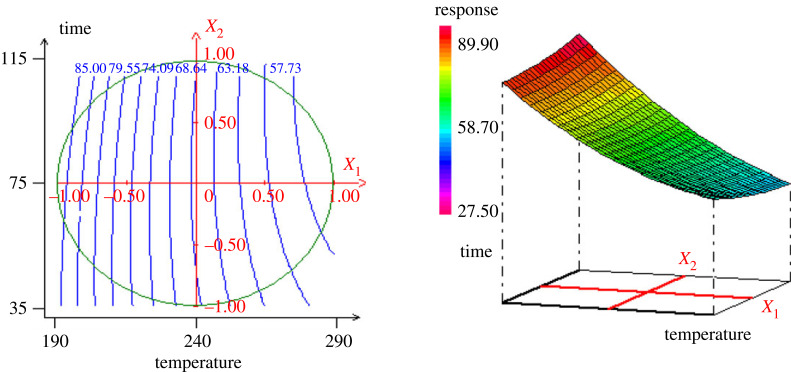
Surface plot and contour plot of the variation of the hydrochar yield (*Y*_1_) versus the temperature and time (*X*_1_, *X*_2_).

**Figure 4.**
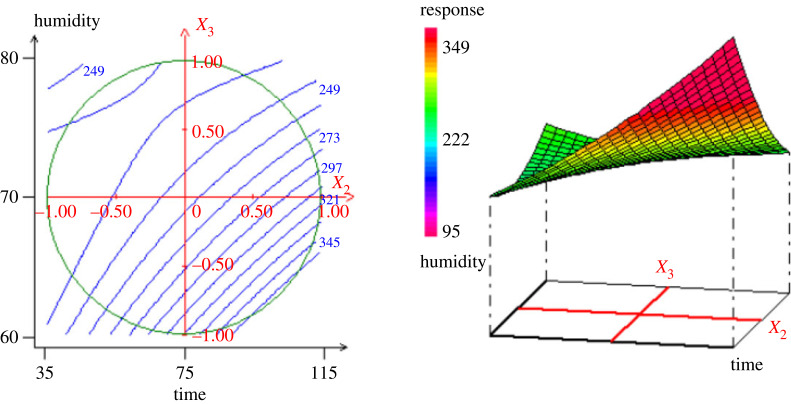
Surface plot and contour plot of the variation of the iodine number (*Y*_2_) versus the time and the humidity (*X*_2_, *X*_3_).

**Figure 5.**
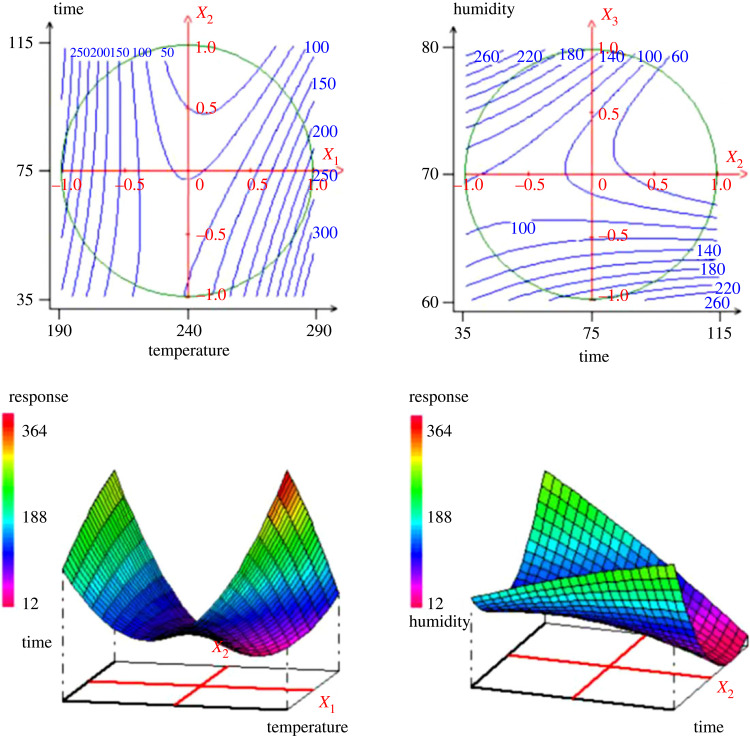
Surface plot and contour plot of the variation of the methylene blue adsorption (*Y*_3_) versus the temperature, residence (*X*_1_, *X*_2_) and versus time and humidity (*X*_2_, *X*_3_).

According to the established model, figures 3–5 show the contour plots and response surfaces curves used to show the most important factors for hydrochar yield.

The figure 3 shows that both temperature and residence time have a substantial influence on the hydrochar yield (*Y*_1_). The analysis of the plot shows that, with the increase in temperature (*X*_1_) from 190°C to 290°C and the residence time (*X*_2_) from 35 to 115 min, the hydrochar yield decreases from 85% to 57.73%. This can be explained by the fact that, as the temperature increases during the hydrothermal carbonization process, there is a release of heteroatoms and volatile compounds (CO_2_, CO, NO, H_2_O, N_2_, SO_2_, organic volatile products) leading to an inevitable loss of mass. The HTC process involves three main reactions—dehydration, condensation and decarboxylation—resulting in a mass loss. From the figure 4, it is observed that the time (*X*_2_) and the humidity (*X*_3_) are the two main influencing factors. Indeed, the amount of iodine adsorbed decreases from 345 to 249 mg g^−1^ with increase in humidity. Since the iodine number gives an indication on the opened microporosity of the material, for the humidity less than the centre point, the increase in humidity promotes micropores formation. The variation of the methylene blue adsorption (figure 5) shows that when the temperature increases from 190°C to 290°C, with the time from 35 to 115 min, the MBN increased from 100 to 300 mg g^−1^. When the time increases from 35 to 115 min with the humidity level of 60% to 80%, the MBN also decreased from 260 to 100 mg g^−1^. These results allow to conclude that the increase in temperature facilitates the opening of the pores on hydrochar, which interestingly increases the adsorption of MB. On the other hand, the increase of humidity is favourable for the formation of micropores as it has also been observed in the adsorption of iodine.

#### 3.3.2. Optimization using model equation

**Figure 6.**
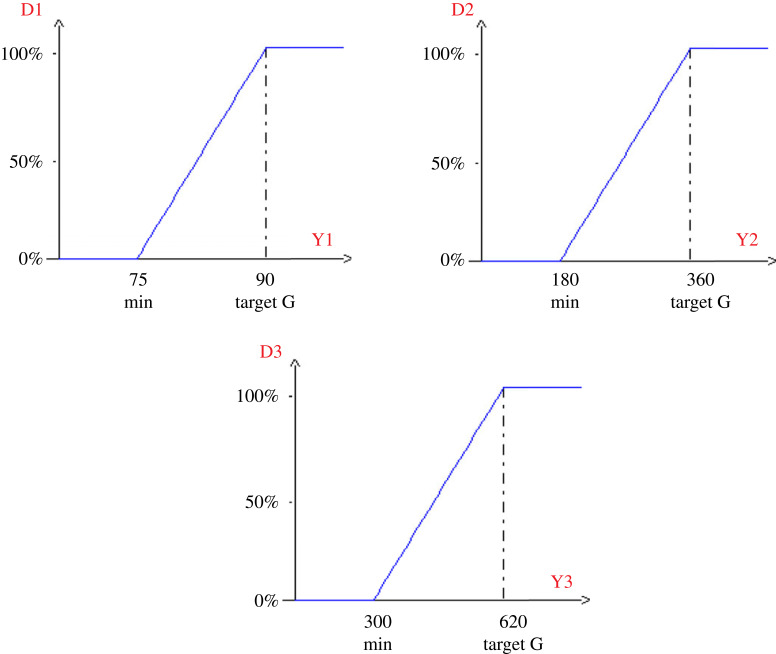
Graphs of the desirability functions for the responses *Y*_1_, *Y*_2_ and *Y*_3_.

**Figure 7.**
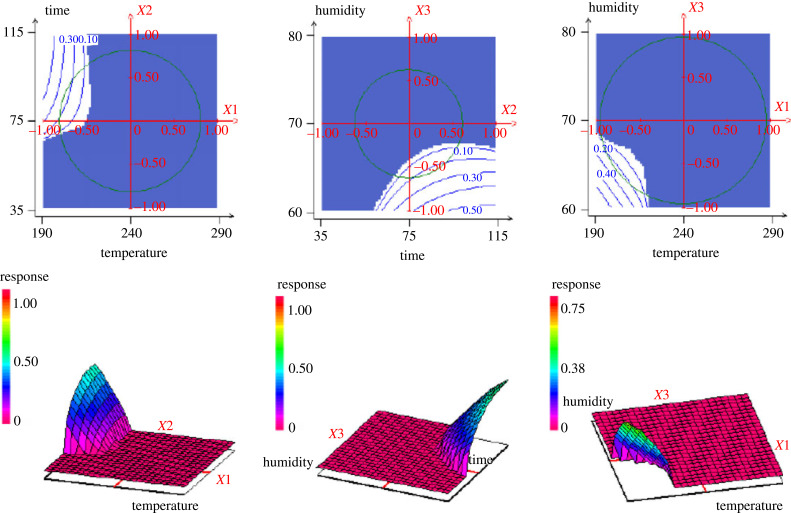
Desired zones of interest of different variation.

In order to find a better compromise that can satisfy all the needs that are the hydrochar in quantity and quality, the desirability function was applied using the NEMROD software. Table 4 gives the optimum characteristics of the hydrochar. The graphs of the desirability functions (figure 6) of the responses show different levels of constraints. The respective minimum and maximum values are: 75.00 to 90.00% for *Y*_1_, 180.00 to 360.00 mg g^−1^ for *Y*_2_ and 300.00 to 620.00 mg g^−1^ for *Y*_3_. The predicted values are: 83.10% for the hydrochar yield, 390.44 mg g^−1^ for iodine number and 259.63 mg g^−1^ for the methylene blue number, which correspond to 53.98%, 28.26% and 44.24% degree of satisfaction for *Y*_1_, *Y*_2_ and *Y*_3_ respectively. The superposition of the surface curves of *Y*_1_, *Y*_2_ and *Y*_3_ helps to identify the optimal zone with the best compromise of desirability. The minimum and maximum values of the predicted desirability are from 34.53 to 45.86. The total desirability of the process is 40.72%, which is satisfactory since this value is within the predicted range. Figure 7 depicts the desired area of interest.

**Table 4.** Characteristics of optimum VTS-HTC. Where di max % represents the degree of maximal satisfaction; di min %, the degree of minimal satisfaction and di% is the degree of satisfaction.

responses	name	values	di %	weights	di min %	di max %
*Y*_1_	yield	83.10	53.98	1	46.45	61.50
*Y*_2_	ION	390.44	28.26	1	24.84	31.69
*Y*_3_	MBN	259.63	44.24	1	35.67	52.81
	desirability		40.72		34.53	46.86

According to its curves (figure 7), the unhatched areas represent the area of interest found. The uncoloured areas (iso response curves) and the raised areas (response surface curves) represent the areas where the optimum is found. These conditions are 195°C, 87.5 min and 66.7% for the temperature, residence time and humidity, respectively. The hydrochar prepared under these conditions has average values of 83.10%, 390.44 mg g^−1^ and 259.63 mg g^−1^ for the hydrochar yield, iodine and methylene blue numbers, respectively.

### 3.4. Characterization of activated hydrochar prepared under optimum condition

**Figure 8.**
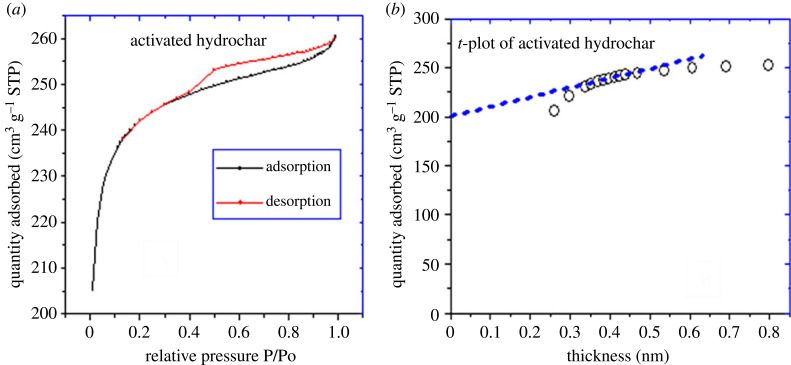
(*a*) Adsorption-desorption isotherms of *N*_2_ and (*b*) *t*-plot for nitrogen adsorbed at 77 K for activated hydrochar obtained at the optimal condition.

A sample of the activated hydrochar obtained under optimum conditions and activated is subjected to characterization. The characterization is an important tool that helps to understand the properties of adsorbent that may affect the removal of micro pollutants in aqueous solution. Figure 8 shows typical N_2_ adsorption–desorption isotherm of the AH obtained from optimal condition. It exhibits the development of both micropores and mesopores. The sample presented greater adsorption capacities at low relative pressures *P*/*P*_o_ < 0.1, indicating the presence of a more developed micropore structure; at relative pressure *P*/*P*_o_ > 0.1 the filling of external pores by capillary condensation is observed. The isotherm of the AH sample is of type II with H3 hysteresis according to the IUPAC, which is associated with a narrow pore size distribution of microporous material. The considerable intensity of hysteresis implies the presence of a network of interconnected pores that open onto the surface via external pores. This can be attributed to the physical activation of hydrochar by steam. Table 5 shows that the activated hydrochar obtained from the optimum has a specific surface area and micropores surface area of 849.160 and 703.269 m^2^ g^−1^ respectively. This shows that the adsorbent prepared is mainly microporous, the observation being attributed to the used of steam during the physical activation.

**Table 5.** Textural parameters of the activated hydrochar.

sample	total surface area BET (m^2^ g^−1^)	micropore area (m^2^ g^−1^)	external surface area (m^2^ g^−1^)	micropore volume (cm³ g^−1^)	total pore volume (cm³ g^−1^)
AH	849.160	703.269	145.891	0.310	0.402

**Figure 9.**
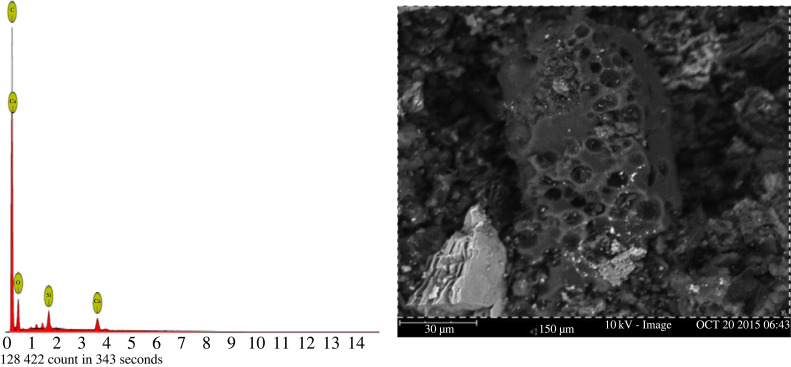
EDX spectrum and SEM micrographs of VTS-HTC obtained under optimal conditions.

The SEM/EDX analysis (figure 9) of the hydrochar obtained in the optimum condition was carried out using an apparatus of the JEOL JSM 6400 brand. It was found that the carbon material exhibits a polydisperse porous structure, made up of aggregates of different sizes and irregular shapes. The porosity is highly developed over the entire surface of the sample with a certain heterogeneity due to the presence of three types of pores. Some white dots are observed from a close observation, attributable to the inorganic composition of precursor. The EDX analysis highlights the elements present on the surface of the hydrochar, consisting mainly of carbon (C) and calcium (Ca).

**Figure 10.**
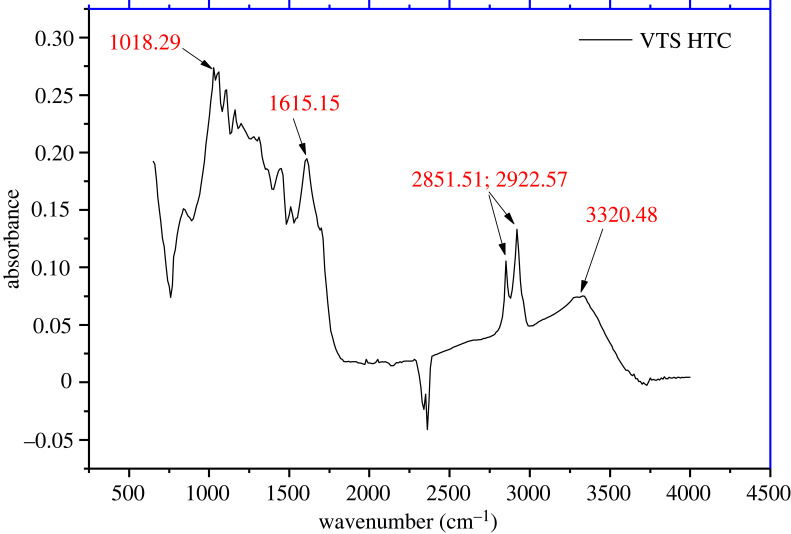
FTIR spectrum of activated VTS-HTC obtained under optimal conditions.

The FTIR spectrum (figure 10) provides valuable information concerning the surface functional groups of the hydrochar material and might better help during its adsorption test. The VTS-HTC spectrum obtained at optimum conditions reveals the existence of several functional groups corresponding to characteristic bands. The adsorption band which appears at 3320 cm^−1^ can be assigned to the OH stretching mode of hydroxyl groups, whereas the absorption band between 2851 and 2922 cm^−1^ corresponds to the C-H function [26]. The absorption peaks around 1615 and 1018 cm^−1^ can be attributed to C-N and C-O functions respectively.

## 4. Conclusion

The response surface methodology allowed to study the simultaneous effect of the temperature of carbonization, residence time and moisture content on the hydrochar preparation based vegetable-tanned leather shavings by hydrothermal carbonization method. Based on the ANOVA, it was observed that the preparation process depends on the residence time and the final hydrochar mass decreases with increase in carbonization temperature. Its efficiency in adsorbing iodine in aqueous medium was found to decrease with increase in humidity of precursor. On the other hand, the methylene blue adsorption was increased under the influence of three parameters studied, whose influence was also found to be both synergetic and antagonistic. The hydrochar micrographs obtained under optimal conditions show mesopores and macropores on the surface which also serve as access to the micropores. The FTIR analysis reveals the presence of functional groups on the hydrochar surface which can serve as adsorption sites. The resulting hydrochar has been found to be a very effective adsorbent for the removal of organic molecules.

## Data Availability

All output files as well as supplementary materials are available from the Dryad Digital Repository: https://doi.org/10.5061/dryad.sf7m0cgbc [30].
